# Double Immunohistochemical Labelling of PRAME and Melan A in Slow Mohs Biopsy Margin Assessment of Lentigo Maligna and Lentigo Maligna Melanoma

**DOI:** 10.3389/bjbs.2024.12319

**Published:** 2024-03-19

**Authors:** R. Salih, F. Ismail, G. E. Orchard

**Affiliations:** St. John’s Histopathology Department, Synnovis Analytics, St. Thomas’ Hospital, London, United Kingdom

**Keywords:** immunohistochemistry, slow Mohs micrographic surgery (SMMS), PRAME, Melan A, lentigo maligna (LM) lentigo maligna melanoma (LMM)

## Abstract

**Introduction:** Lentigo maligna (LM) and lentigo maligna melanoma (LMM) predominantly affect the head and neck areas in elderly patients, presenting as challenging ill-defined pigmented lesions with indistinct borders. Surgical margin determination for complete removal remains intricate due to these characteristics. Morphological examination of surgical margins is the key form of determining successful treatment in LM/LMM and underpin the greater margin control provided through the Slow Mohs micrographic surgery (SMMS) approach. Recent assessments have explored the use of immunohistochemistry (IHC) markers, such as Preferentially Expressed Antigen in Melanoma (PRAME), to aid in LM/LMM and margin evaluation, leveraging the selectivity of PRAME labelling in malignant melanocytic neoplasms.

**Methods:** A Novel double-labelling (DL) method incorporating both PRAME and MelanA IHC was employed to further maximise the clinical applicability of PRAME in the assessment of LM/LMM in SMMS biopsies. The evaluation involved 51 samples, comparing the results of the novel DL with respective single-labelling (SL) IHC slides.

**Results:** The findings demonstrated a significant agreement of 96.1% between the DL method and SL slides across the tested samples. The benchmark PRAME SL exhibited a sensitivity of 91.3% in the SMMS specimens and 67.9% in histologically confirmed positive margins.

**Discussion:** This study highlights the utility of PRAME IHC and by extension PRAME DL as an adjunctive tool in the assessment of melanocytic tumours within staged excision margins in SMMS samples.

## Introduction

Lentigo maligna (LM) is a unique subtype of melanoma *in situ* (MIS), distinguished by its lentiginous growth pattern observed in chronically sun-damaged skin. If an LM lesion becomes invasive, it is no longer confined to the epidermis and can no longer be considered MIS. This invasive form of LM is subsequently referred to as Lentigo Maligna Melanoma (LMM), which acquires a comparable prognosis to other forms of invasive melanomas when comparing Breslow thickness [1].

The histologically assessed surgical removal of the lesion is the primary form of treatment with both wide local excision (WLE), and more specialised staged excision (SE) approaches such as the Mohs Micrographic Surgery (MMS) technique, having been described [2]. However, prior studies have shown that the superior margin control expressed through specialised SE approaches such as MMS, proves to be superior to traditional WLE methods, when treating LM/LMM lesions of patients that present on the head and neck [3, 4]. This is evident in the lower local recurrence rates of 0.61% by MMS when compared to 7.8% by WLE when used to treat cutaneous head and neck melanomas [4]. Furthermore, only 1% of total margins are evaluated through WLE with subsequent vertical sectioning, which is in comparison to the 100% complete demonstration of both deep and peripheral margins offered by MMS [5]. The superior margin control offered by MMS, coupled with the tailored-to-tumour surgical removal of tissue allowing for greater cosmetic consideration, makes the MMS approach particularly suited for treating LM and LMM patients presenting on the head and neck regions [5]. This has led to MMS becoming the standard of care for LM patients, in particular when manifesting in the most common cosmetically-sensitive regions such as the head and neck [2].

In the case of in-situ melanomas such as LM, a variant of the MMS technique known as the “slow” Mohs procedure is typically employed [5, 6]. This alternate method utilises the same lesional mapping and staged surgical approach to traditional frozen sectioning MMS, but with the addition of permanent formalin-fixed paraffin-embedded (FFPE) processing for each Mohs layer, forgoing the use of frozen sections entirely. This longer FFPE excursion is essential for providing the gold standard morphological visualisation of melanocytic lesions that is free from the interpretational and artefactual challenges that is often associated with frozen sections [5]. In addition, routine immunohistochemistry (IHC) adjunct labelling can also be used with FFPE samples in order to aid in LM/LMM margin assessment by reporting pathologists.

A number of IHC “melanocytic markers,” such as SOX10 and Melanoma antigen recognised by T cells (MART-1 or Melan A), have been delineated to directly detect and visualise melanocytes in the skin through differing labelling patterns, both nuclear and cytoplasmic respectively. These markers can potentially facilitate the diagnosis of LM or the assessment of residual LM in surgical margins within the sun-damaged skin setting. Both SOX10 and Melan A can be used to determine a prime parameter; the cellularity or density of melanocytes within slow Mohs sections, which can help facilitate residual LM/LMM identification and by extension, margin clearance [7].

It is important to note however that markers such as SOX-10 and Melan A, whilst they have their usage in LM studies, they both lack the ability to differentiate malignant from benign melanocytes. This labelling of “all” melanocytes, significantly limits their use in differentiating LM from its mimics [8]. However, Preferentially expressed Antigen in MElanoma (PRAME), has been the subject of intense study within the last 5 years, having shown promise in this regard [7–17]. Studies have showed differential PRAME expression within benign and malignant melanocytes, showing high expression in a variety of malignant melanocyte populations—in particular, melanoma and in-situ melanomas such as LM/LMM, with contrasting little to no expression in benign entities such as melanocytic nevi [8–11]. This denotes a differential PRAME expression and specificity towards malignant melanocytes, as opposed to traditional melanocyte markers such as Melan A and SOX-10, that do not discriminate and label all melanocyte populations, both benign and malignant, in varying labelling profiles.

A Key study by Lezcano et al. (2018) sought to evaluate the utility of PRAME IHC as a potentially useful adjunct tool for the assessment of melanocytic tumours, showing early yet promising evidence for PRAME IHC as a useful adjunctive tool within both melanoma and margin assessment studies [9]. Since then, many others have sought to evaluate PRAME using a variety of approaches and subject matter, seeking to further elucidate the strengths, limitations and pitfalls of PRAME IHC focusing on the differentiation of benign and malignant melanocytic entities [7–18].

Most congruously, some recent studies have explored the potential for PRAME nuclear labelling, to be used in various double-labelling methods, combined with cytoplasmic HMB-45 or Melan A, in order to further enhance the utility of PRAME within melanocytic investigations [7, 8, 11–13].

This study will seek to determine the utility of PRAME IHC by establishing the sensitivity of PRAME SL within the novel application of LM/LMM SMMS biopsies. The study will also seek to demonstrate and directly compare a novel PRAME/Melan A DL protocol to the aforementioned PRAME SL benchmark for agreement. This is to determine whether PRAME/Melan A DL IHC can be used as a useful alternative to PRAME SL IHC, providing additional information through cytoplasmic Melan A labelling, within the same section. Once established, the novel DL could therefore be used as an adjunct to H&E routine morphology within SMMS LM/LMM margin assessment.

## Materials and Methods

### Case Selection and Histological Characteristics

Records from the dermatopathology Laboratory at St. John’s Institute of Dermatology were searched for Slow Mohs excision biopsies from 2020 to 2022 for cases diagnosed with LM/LMM. A total of 23 anonymised LM/LMM slow Mohs cases were selected. Of all 23 cases, the histological characteristics of 15/23 de-bulk sections and 22/28 positive margin sections were available within the pathology reports examined. Within each of the applicable reports examined, the use of several histological features supporting LM/LMM were tabulated and are summarised in Table 1. The 3 highest histological features in support of LM found within the reports to describe both de-bulk and positive margins respectively were pleomorphism/atypia (100%/100%), increased number of melanocytes (86.7%/91%) and confluency (60%/77.3%). Other features such as adnexal involvement (33%/31.8%) and nest formation (20%/22.27%) were less frequent. From these cases, a total of 51 formalin-fixed paraffin-embedded (FFPE) blocks, comprised of 23 de-bulk and 28 histologically confirmed positive margins were incorporated within the study. For larger cases, more than one histologically positive margin FFPE block was used. All Blocks were labelled with letters corresponding to the respective sections of each histology report for histological correlation. Given the retrospective nature of the study, each of these blocks were stained with an initial H&E to ensure block viability and relevance to the anonymised histology reports provided, which were checked by Consultant BMS GEO. All further IHC tests, Melan A SL IHC, PRAME SL IHC, and PRAME/Melan A IHC DL were performed on serially sectioned slides for direct comparison.

**TABLE 1:** Summary of the histological characteristics of LM/LMM cases examined.

H&E morphological criteria used for LM/LMM diagnosis	De-bulk	Positive margins *N* = 22
	*N* = 15	
Aytpia/pleiomorphism	15 (100%)	22 (100%)
Increased number of melanocytes	13 (86.7%	20 (91%)
Irregular pigment deposition	1 (6.7%	0
Pagetiod spread	1 (6.7%)	2 (9.1%)
Epidermal atrophy/effacement	2 (13.3%)	2 (9.1%)
confluency	9 (60%)	17 (77.3%)
Nest formation	3 (20%	5 (22.7%)
Adnexal involvement	5 (33.3%)	7 (31.8%)

### Test Selection and Technical Considerations

PRAME/Melan A DL IHC combines Melan A cytoplasmic labelling to highlight all melanocyte populations within a section, with the added nuclear labelling of PRAME to label “malignant” melanocytes. This allows for the visualisation of dual-labelled malignant melanocytes alongside benign melanocytes labelled with Melan A in the same section.

At present, there is no existing literature regarding the utilisation of PRAME/Melan A DL IHC in the context of margin assessment for SMMS biopsies within LM/LMM. As a result, the current study has utilised the established PRAME SL IHC as the benchmark for clinical correlation, alongside the novel DL method in parallel for establishing agreement between both methods. Melan A SL IHC were run in parallel in order to validate the Melan A component of the DL.

In addition to IHC stains, H&E staining was also used for demonstrating the morphology and histological criteria of LM/LMM.

**FIGURE 1:**
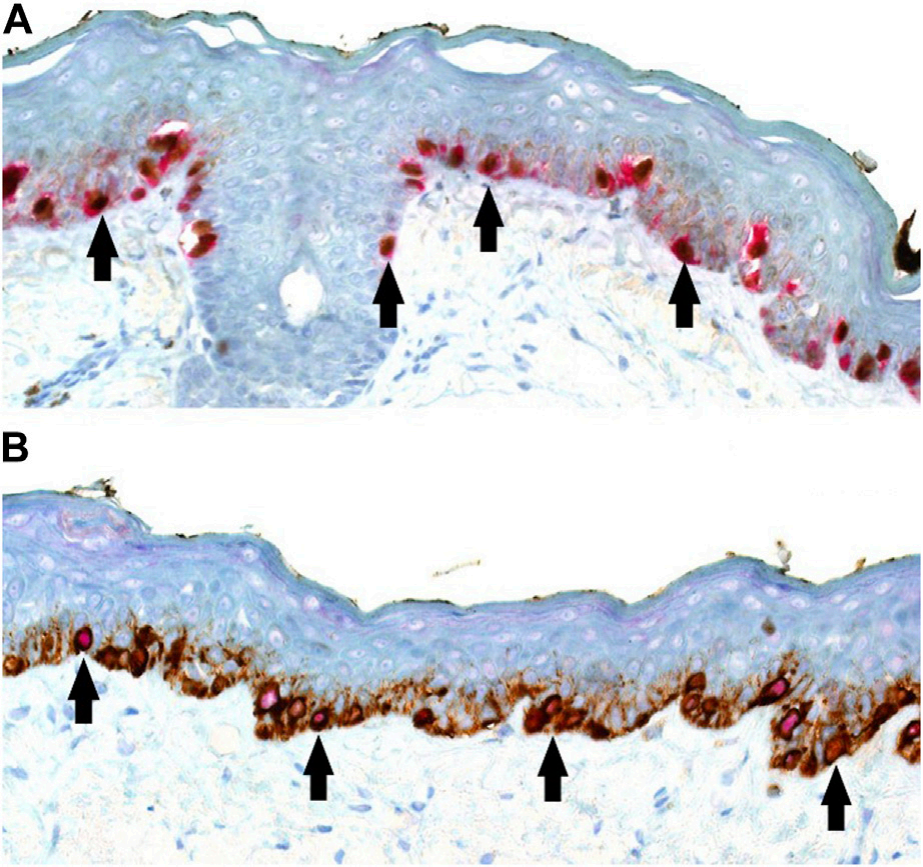
**(A,B)** PRAME/Melan A double labelling within LM control sections (×20 magnification) demonstrating possible visual outcomes depending on chromogen selection. **(A)** Alternative PRAME nuclear labelling in brown, combined with Melan A cytoplasmic labelling in red. **(B)** Showing the selected method of PRAME with red nuclear labelling, combined with brown Melan A cytoplasmic labelling of respective LM melanocytes. For both chromogen arrangements, double-labelled melanocytes are shown with arrows.

All slides and tests were generated in a serial manner for accurate interpretation and juxtaposition of photography. An initial set of positive control material was used to select the combination of chromogen for IHC DL based on visual performance and contrast, with the latter combination of red nuclear PRAME labelling with brown cytoplasmic Melan A labelling, being taken forward for the study (Figure 1B). Both the SL Melan A (brown) and PRAME (red) used the same respective chromogen for direct comparisons to be made.

### Histological Material Preparation

All anonymised FFPE blocks used within the study were sectioned by author RS over the course of 4 batches which were carried out on 4 consecutive days with key factors controlled for, such as, water bath set to 37°C, same equipment used each day, and same batch numbers for reagents used. This was to reduce batch-to-batch variability.

All slides were sectioned at 3 µm and picked up on either TOMO^®^ IHC adhesive slides (Solmedia Ltd., Shrewsbury, United Kingdom) for IHC labelling with positive control material or on SuperFrost®Plus Adhesion Slides (VWR^®^, Leicestershire, United Kingdom) for H&E sections which will have been consistent to the original H&E slides for reporting.

H&E staining was performed using the automated Leica Autostainer XL [Leica Microsystems (UK) Ltd. Milton Keynes, United Kingdom], using a commercially available Harris’ haematoxylin and a 1% aqueous Eosin [Leica Microsystems (UK) Ltd. Milton Keynes, United Kingdom]. H&E staining was carried out using the same routine protocol that is used within the laboratory.

Cut slides for IHC were left to dry in a 37°C oven for 10 min before being transferred to a 60°C oven to bake for 1 h. Slides were then labelled according to the anonymised format given and loaded onto the IHC labelling platform on the same day that each batch was produced.

### IHC

A total of 153 IHC slides were performed, 51 slides per each IHC test. All slides were generated using the Roche Ventana Benchmark ULTRA IHC automated immunostaining platform (Roche Diagnostics Limited, West Sussex, United Kingdom). The same IHC machine was used throughout the study in order to prevent any potential variability that may occur during the IHC procedure.

Melan A IHC was carried out using the Roche ready-to-use (RTU) anti-MART-1/Melan A (A103 clone, from Roche Diagnostics Limited, West Sussex, United Kingdom) mouse monoclonal primary antibody. PRAME IHC was carried out using anti-PRAME (QR005 clone) RTU rabbit monoclonal primary antibody which was obtained from AnatoPath limited (, Saxmundham, England). Both primary antibodies and their subsequent IHC protocols were validated well in advance by GEO and FI. The DL protocol was developed and optimised in-house prior to use in this study. All three IHC protocols, Melan A SL, PRAME SL and PRAME/MelanA DL followed manufacturer recommendations where possible. This involved the same antigen retrieval step using commercially available Roche (Roche Diagnostics Limited, West Sussex, United Kingdom) ULTRA Cell Conditioning Solution 1 (ULTRA CC1) for heat-mediated antigen retrieval, coupled with polymer based detection kits also supplied by Roche (Roche Diagnostics Limited, West Sussex, United Kingdom) for both brown (OptiView DAB IHC Detection Kit, Ref: 760-700) and red chromogen (ultraView Universal Alkaline Phosphatase Red Detection Kit, Ref: 760-501) respectively. Counterstain of IHC slides were all carried out using Roche (Roche Diagnostics Limited, West Sussex, United Kingdom) Hematoxylin II which is designed for FFPE tissues on the BenchMark IHC automated platform.

A known positive control was also added to each IHC slide as an extra layer of validation.

### Evaluation of DL and Scoring Criteria for PRAME and Melan A

The technical evaluation of the DL was done through direct qualitative comparison to each respective single IHC slide, PRAME SL and Melan A SL, to ensure that there was no artefactual, or potential pitfalls in the appearance of the DL—namely focusing on false loss or gain or obscurity in PRAME or Melan A components when juxtaposed. Comparison of IHC stains as well as the scoring of PRAME, were done through visual assessment under a light microscope by a Consultant BMS G.E.O.

Scoring criteria of the PRAME SL IHC benchmark within the study was based on the percentage of PRAME positive tumour cells that were present within the lesion examined. Thus 0—negative, +1 (1%–25%), +2 (26%–50%), +3 (51%–75%), +4 (>75% diffuse/positive) [9]. Only lesions that exhibited more than 75% (+4 labelling) of PRAME positive lesional cells were scored as positive. 0% indicates a complete absence of any PRAME labelling and was therefore negative. Intermediate labelling, +1, +2, +3, was also interpreted as negative within the study, which was in line with prior investigations [7–11, 13–15]. In summary PRAME IHC slides were scored as either positive (+4 diffuse) or negative (0, +1, +2, +3) within the study when assessing the sensitivity of PRAME within SMMS biopsies. However, when assessing the novel DL technique to the PRAME SL, each slide was assessed for agreement based on the specific labelling of PRAME that was seen, 0, +1, +2. +3 and +4 respectively.

Melan A SL IHC was used as the comparative benchmark for the Melan A component of the novel DL. Melan A IHC interpretation was based on the positive or negative presence (+ve) or absence (−ve) of cytoplasmic melanocyte labelling within SMMS biopsies respectively.

### Statistical Analysis

Statistical analysis and measurement of agreement between the novel DL and SL was performed using SPSS software (IBM Corp. Released 2021. IBM SPSS Statistics for Windows, Version 29.0. Armonk, NY: IBM Corp), utilising the Kappa statistic.

## Results

### Evaluation of PRAME SL IHC in Slow Mohs Sections

Results of the PRAME SL IHC can be found in Table 2. 21/23 LM de-bulk sections that were used for PRAME SL IHC were found to be diffusely positive (>75% threshold). Of the remaining 2/23 de-bulk sections one exhibited an intermediate +3 PRAME labelling, with the other section showing absent 0 PRAME immunoreactivity. The same PRAME negative (0) de-bulk section had PRAME negativity (0) at both histologically positive margins. All sections that showed 0 immunoreactivity for PRAME (0) were all derived from the same case. By using +4 as the positivity threshold for PRAME, a PRAME IHC sensitivity of 91.3% was obtained with respect to de-bulk sections examined (*n* = 23). A lower PRAME sensitivity of 67.9% (19/28) was observed with respect to PRAME SL IHC labelling at the margins (*n* = 28) using the same +4 positivity criteria. Of the 9/28 histologically confirmed positive slides that failed to meet the +4 threshold for positivity, 2/28 were the aforementioned slides with 0 labelling, 3/28 exhibited +1 labelling, 2/28 showed +2 labelling and finally, 2/28 demonstrated +3 labelling. The combined sensitivity for PRAME including both de-bulk and positive margins, 40/51, was 78.4%.

**TABLE 2:** PRAME SL IHC labelling results obtained from the analysis of 23 LM/LMM slow mohs cases. Cases were broken down into 23 de-bulk sections (one for each case) and a collection of 28 histologically positive margins across the 23 cases incorporated. PRAME was only concluded as positive or “diffuse” if +4 (>75% of lesional cells) threshold was met.

LM positive sections *n* = 51	PRAME IHC labelling criteria denoted as a percentage of labelled lesional tumour nuclei				
	Negative 0 (0%)	+1 (1%–25%)	+2 (25%–50%)	+3 (51%–75%)	Positive (>75%) “diffuse”
De-bulk sections (*n* = 23)	1^a^	0	0	1	21
Positive margin sections (*n* = 28)	2^a^	3	2	2	19

^a^ One case examined was reported as LM histologically but was negative for PRAME IHC in both the de-bulk and two margin sections.

### Evaluation of PRAME/Melan A IHC DL

There was an overall substantial agreement between the novel DL and the PRAME SL of 96.1% (Kappa = 0.895 with *p*-value = <0.001). All Melan A (*n* = 51) IHC seen between the novel DL method and the control Melan A SL were 100% concordant with all slides showing positive cytoplasmic labelling of melanocytes where present. The 2/48 non-concordant DL slides were derived from histologically positive margins that showed weak +1 and +2 PRAME immunoreactivity on the respective PRAME SL slides. Both respective DL slides showed no PRAME nuclear immunoreactivity when compared to the PRAME SL control. Comparison of the novel DL is summarised in Table 3.

**TABLE 3:** Comparison of PRAME/Melan A DL to PRAME SL IHC and Melan A SL IHC. Each slide is compared and categorised according to labelling outcomes between the SL and DL.

SL IHC results	PRAME					Melan A
	Immunoreactivity absent - 0	+1	+2	+3	+4	Positive
	*N* = 3	*N* = 3	*N* = 2	*N* = 3	*N* = 40	*N* = 51
DL IHC results	3	2	1	3	40	51
DL IHC concordance to SL IHC (%)	96.1% (49/51)					100% (51/51)
*N* = 51						

### Qualitative Comparisons of Novel DL to SL and Morphological H&E

**FIGURE 2:**
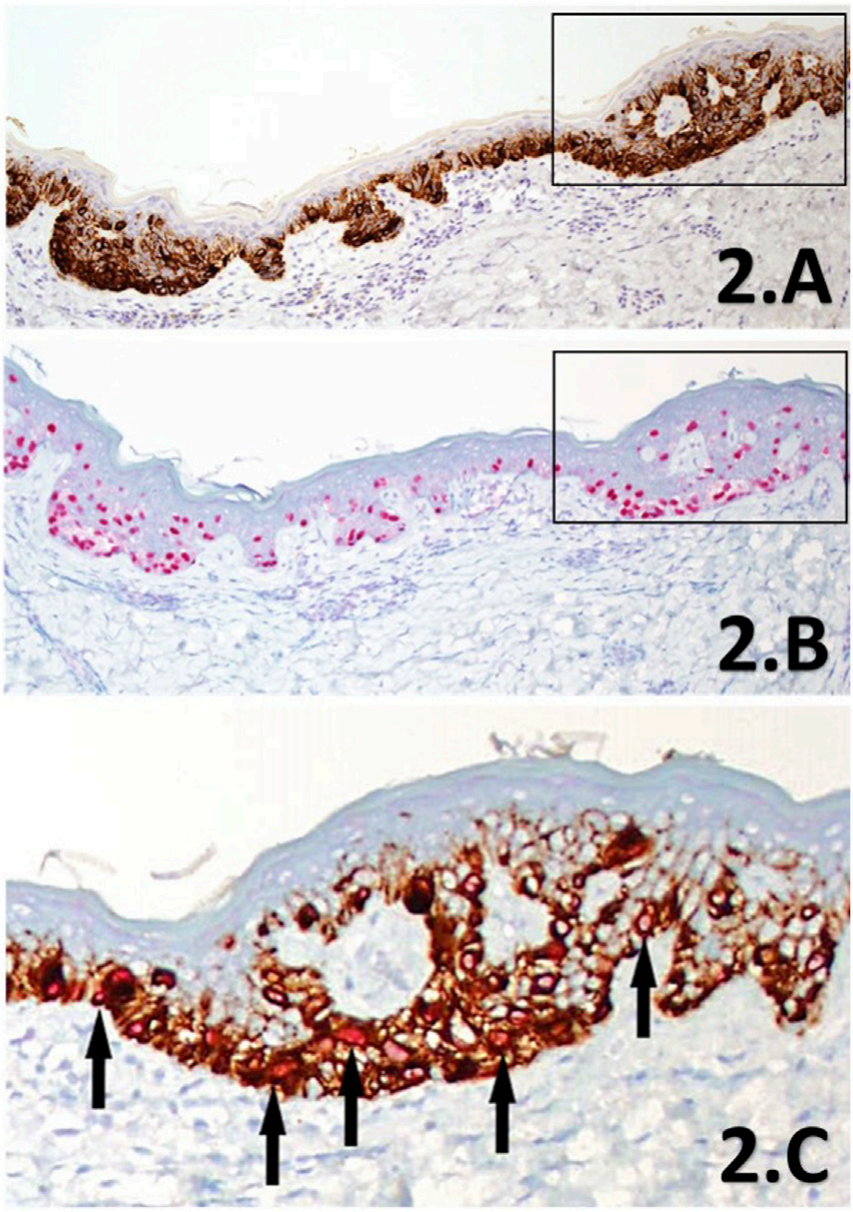
**(A–C)** IHC sections of a SMMS LM de-bulk specimen derived from the left forehead. Melan A IHC SL **(A)** showing intense cytoplasmic labelling of melanocytes within the junctional component in brown chromogen, highlighting the increased proliferation of intraepidermal melanocytes in a lentiginous pattern, with accompanying nest formation (×10 magnification). PRAME IHC SL **(B)** shows diffuse nuclear labelling with almost all lesional melanocytes (>75%) within the junctional component are labelled with the red chromogen (×10 magnification). The black rectangles highlight the direct region of the corresponding magnified area within the PRAME/Melan A DL IHC **(C)** section for comparison to the SL counterparts. The DL (×20 magnification) shows clear double-labelling (black arrows) of almost all junctional melanocytes with bothPRAME red nuclear labelling, and brown cytoplasmic labelling of the cytoplasmic Melan A. Almost all melanocytes can be seen to be labelled with PRAME (both the DL and SL) in this select region showing strong concordance between the three IHC methods, providing further support in favour of LM.

**FIGURE 3:**
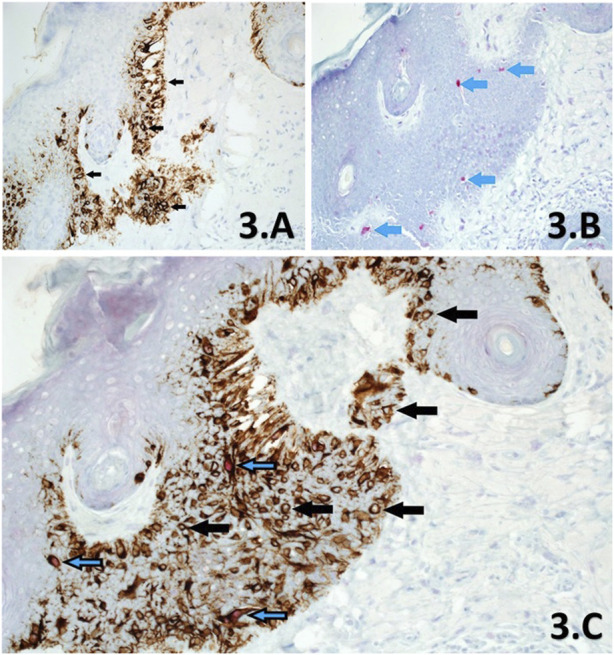
**(A–C)**. Sections of skin derived from a previously reported residual LM, from a slow Mohs excision margin taken from the left side of nose. Melan A SL IHC **(A)** shows significant proliferation of melanocytes at the basal layer through cytoplasmic labelling of all melanocytes within the section, showing dendritic processes (×10 magnification). Examples of melanocytes are shown (black arrows) with clear nuclei. PRAME SL IHC **(B)** shows +1 red nuclear labelling of a few (<10%) melanocytes (blue arrows) within the same area of the lesion (×10 magnification). This section is interpreted as PRAME negative. PRAME/Melan A DL IHC **(C)** at ×20 magnification, shows both populations of melanocytes within the same area of the lesion, exhibiting labelling that is in agreement to both respective SL counterparts **(A,B)**. Note that PRAME negative melanocytes (black arrows) make up >90% of the lesion and lack any nuclear labelling or artefactual distortions as a result of double-labelling, which is comparable to the Melan A SL **(A)**. Similarly, PRAME positive melanocytes are demonstrated through DL with a red nuclear component (blue/black arrows). DL melanocytes comprised <10% of the lesional population within the same area, which is in direct accordance to the PRAME SL IHC control **(B)**. The DL is also interpreted as PRAME negative.

**FIGURE 4:**
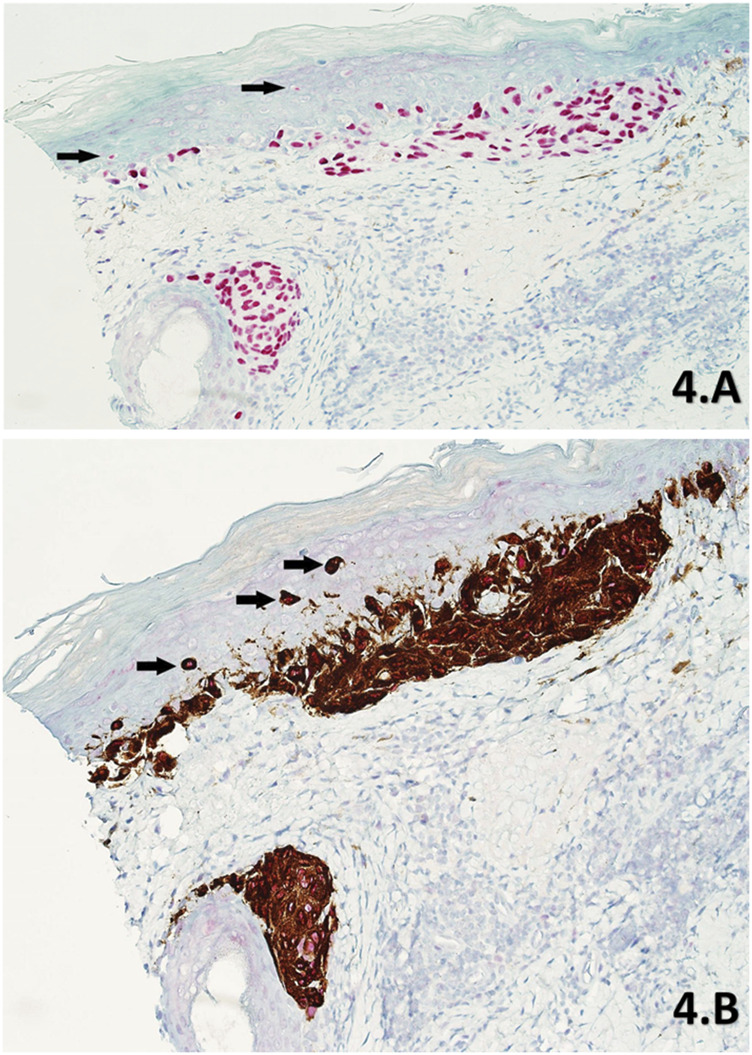
**(A,B)** Skin sections of a part of residual LM from a slow Mohs de-bulk biopsy labelled with PRAME SL IHC **(A)** and PRAME/Melan A DL IHC **(B)** respectively (×20 magnification). Both sections show diffuse +4 positive red nuclear labelling of atypical melanocytes within the lesion. The lesion is comprised of atypical melanocytes that have formed densely packed nests at the base of the epidermis and nearby hair follicle. The DL **(B)** also shows intense cytoplasmic Melan A labelling of dendritic processes within these nests, with subsequent PRAME red nuclear labelling being detectable at higher magnifications. Upward pagetoid migration of atypical melanocytes (Black arrows) is seen more clearly in the DL **(B)** than the SL **(A)**.

Following qualitative assessment by G.E.O, several superimposed and annotated, concordant photographs were taken to provide direct qualitative comparison between the novel DL and the PRAME SL IHC and Melan A SL IHC controls Figures 2–4. These images were selected for performance and technical aspects relating to the DL method.

**FIGURE 5:**
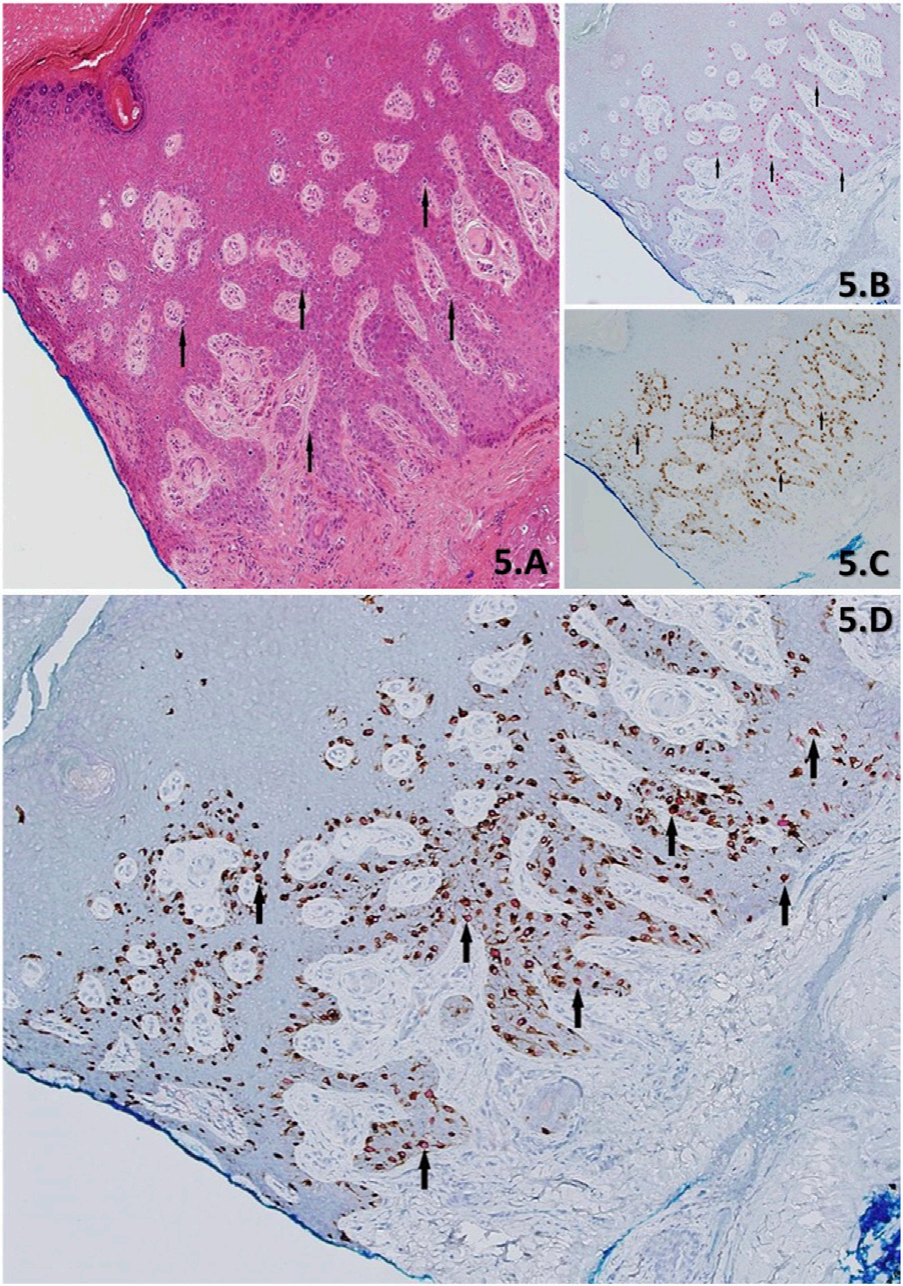
**(A–D)** Demonstration of skin with residual LM from a stage-II slow Mohs biopsy, using H&E **(A)**, PRAME IHC SL **(B)**) Melan A IHC SL **(C)** and PRAME/Melan A DL IHC **(D)**. H&E Section shows acral skin with cross-cutting, with an eccentrically located lesion that extends into the blue inked peripheral margin. The lesion is comprised of lentiginous melanocytes (black arrows) exhibiting severe cytological atypia. IHC show in support of residual LM and is denoted by; >75% (+4) red nuclear labelling of “atypical” melanocytes (black arrows) within the lesion by the PRAME SL **(B)**, concordant >75% (+4) of dual-labelled “atypical” melanocytes (black arrow) of PRAME with red nuclear labelling and Melan A brown cytoplasmic labelling DL **(D)**, and Melan A SL **(C)** brown cytoplasmic labelling of melanocytes (black arrow) showing the entire melanocyte population. All images taken at ×10 magnification.

**FIGURE 6:**
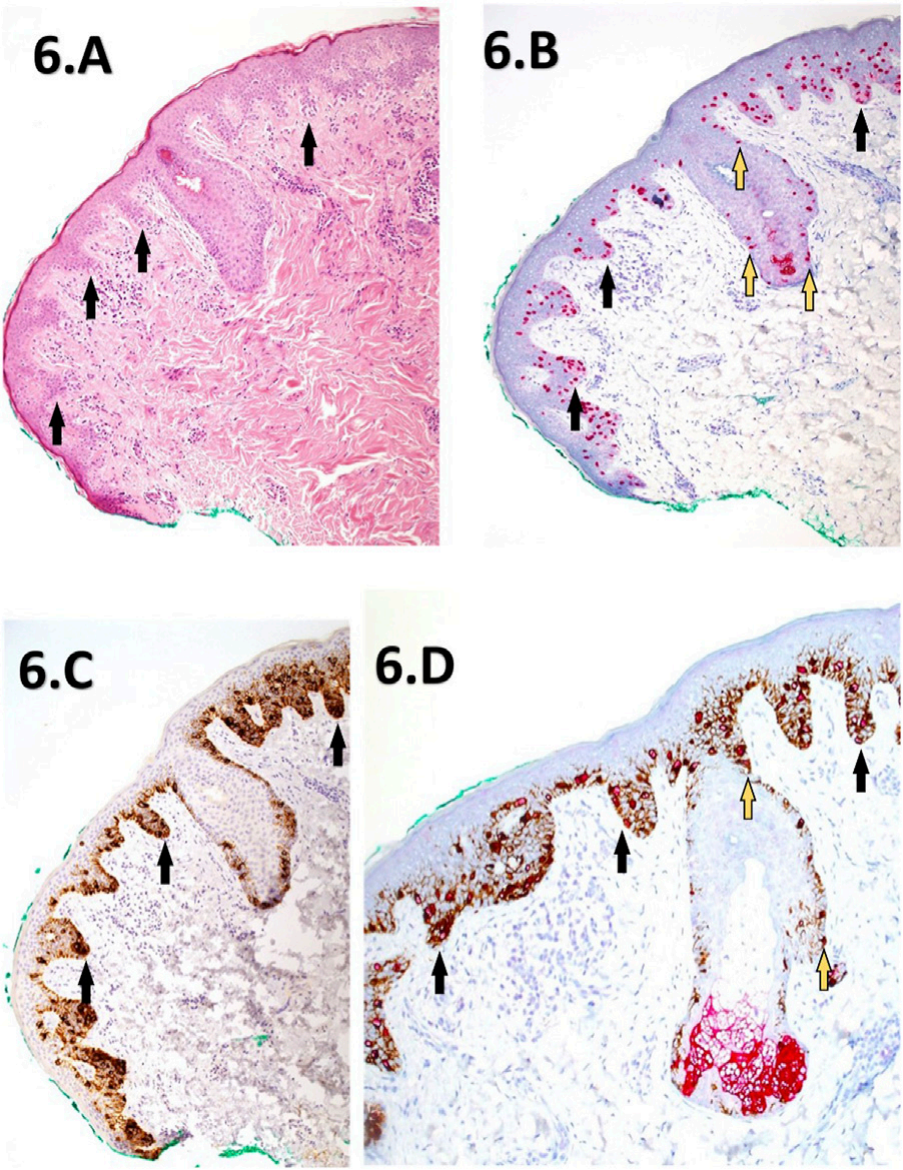
**(A–D)** Corresponding images depicting a slow Mohs excision margin of a previously reported residual LM from the right cheek. The images include H&E staining **(A)** at (×10 magnification), PRAME SL IHC **(B)** at ×10 magnification, Melan A SL IHC **(C)** at ×10 magnification, dual labelling (DL) at ×20 magnification **(D)**. The H&E section **(A)** reveals mild hyperkeratosis and elongation of rete ridges, with atypical melanocytes exhibiting lentiginous and focal nested proliferations (black arrows) at the dermal-epidermal junction. Descent down the adnexal hair follicle epithelium is also noted. PRAME SL IHC **(B)** displays strong +4 diffuse positivity of melanocytes within the lesion, extending into nearby adnexal structures (yellow-blue arrows). Melan A SL IHC **(C)** shows cytoplasmic labelling of a lentiginous proliferation of melanocytes within the junctional component, featuring focal nests (black arrows). The DL image **(D)** demonstrates similar +4 PRAME nuclear labelling, with most melanocytes dual-labelled with the Melan A cytoplasmic component. The IHC sections collectively support the presence of residual LM extending to the green peripheral margin.

**FIGURE 7:**
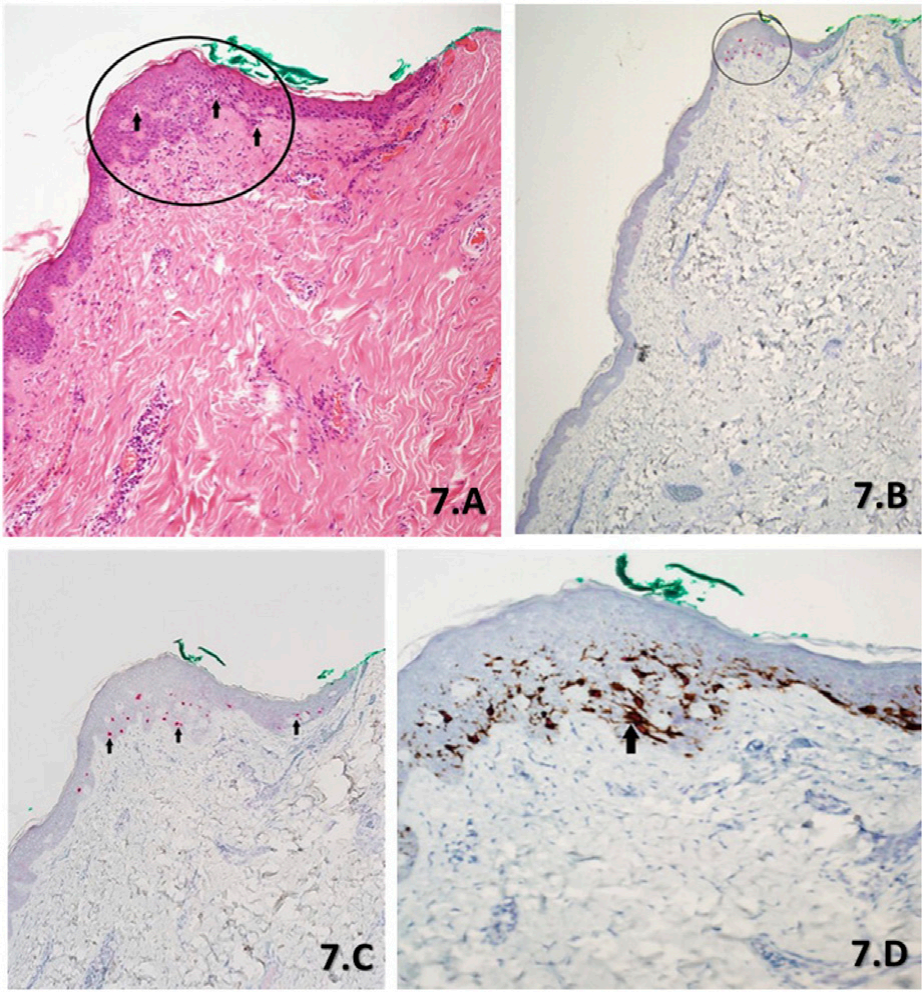
**(A–D)**. Serial sections of a slow Mohs margin biopsy stained with H&E **(A)** at ×10 magnification, PRAME SL IHC **(B)** at ×4 magnification, PRAME SL IHC **(C)** at ×10 magnification and DL **(D)** at ×20 magnification. The H&E **(A)** morphology as per the report showed lentiginous hyperplasia of non-contiguous melanocytes with focal cytological atypia throughout the epidermis. However, there is a focal proliferation of melanocytes with severe cytological atypia (black arrows), 0.5 mm from the green peripheral margin (black circle). These atypical melanocytes appear contiguous and are suspicious for a small focus of residual LM. PRAME SL IHC ×4 magnification **(B)** and PRAME SL IHC ×10 magnification **(C)** draw attention to the focally located atypical melanocytes with positive +4 PRAME nuclear labelling (red) when accounting for the small focal area. The DL **(D)** shows almost all melanocytes as depicted with brown cytoplasmic labelling of Melan A within the focal area, to have corresponding PRAME nuclear labelling to the SL **(B,C)**. Additionally, a group of contiguous atypical melanocytes can be seen (black arrow).

**FIGURE 8:**
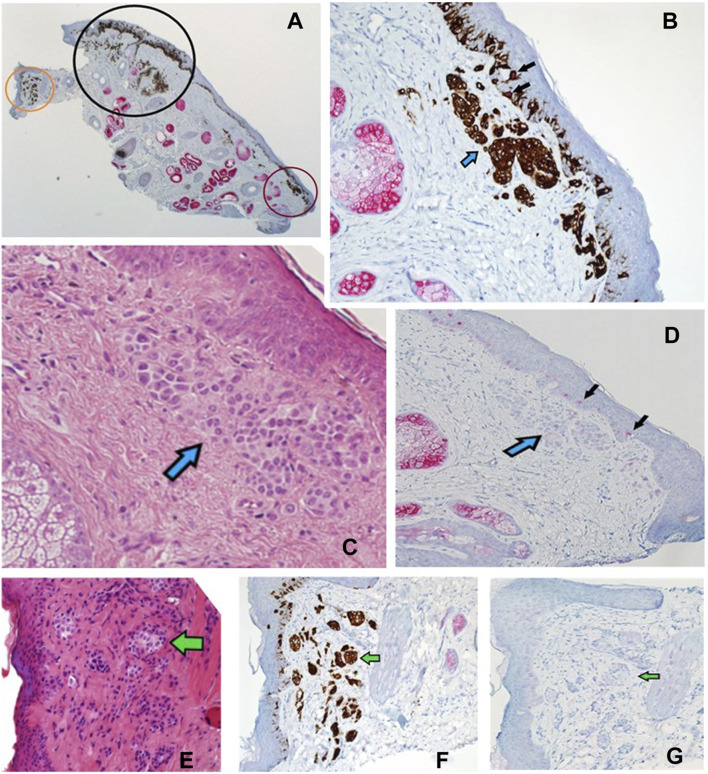
**(A–G)** Sections of a Mohs de-bulk specimen reported as a LM with underlying naevus. PRAME/Melan A DL **(A)** at ×2 magnification showing the overall localisation of melanocytic proliferations (brown chromogen) into three distinct areas, a left non-involved region (orange circle), a middle region with LM component (black circle), and a right non-involved region (red circle). Sections B-D show the red circled region at higher power. **(B)** Shows DL labelling (×20 magnification) that highlights both junctional and dermal (blue arrow) melanocytic components with a lack of PRAME labelling in most cells. Only two melanocytes are seen with dual-labelling (black arrows), overall suggestive of a benign process. **(C)** Shows H&E (20x magnification) staining depicting a large dermal melanocytic proliferation (blue arrow) that amounts to a benign dermal nevus that is found within the red circled region. **(D)** Shows PRAME SL IHC (×10 magnification) that is negative for PRAME within the dermal nevus (blue arrow) but some weak positivity (+1) within the epidermis (black arrows). **(E)** Shows H&E (×20 magnification) staining of the orange circled region depicting another dermal naevus proliferation (green arrow). This is highlighted by the DL (×20 magnification) within the same orange region **(F)** showing only Melan A cytoplasmic labelling without any PRAME (green arrow). **(G)** PRAME SL IHC (×20 magnification) showing lack of labelling (green arrow) in the orange region, suggestive of a benign dermal naevus.

**FIGURE 9:**
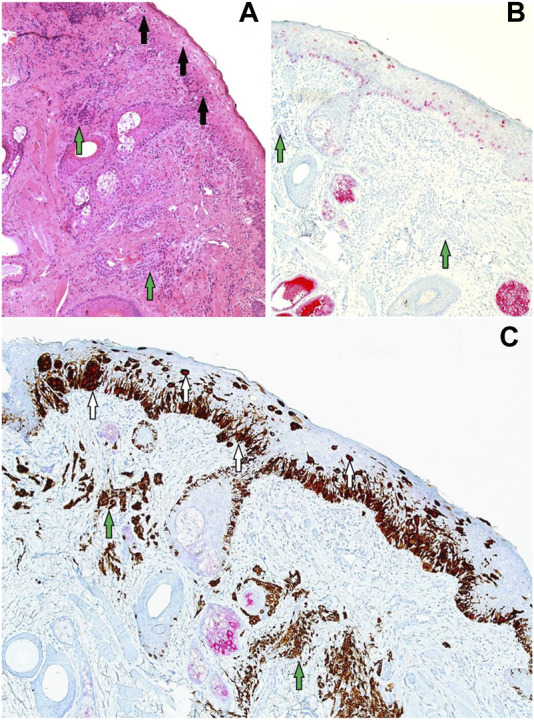
**(A–C)** Sections (all at ×20 magnification) corresponding to the black circled region from Figure 8. **(A)** H&E showing a proliferation of contiguous atypical melanocytes along the junctional component, arranged in a lentiginous pattern with marked upward pagetiod spread (black arrows). The features of the overlying junctional component are in keeping with LM. There is also an underlying melanocytic proliferation with suggestive features of a dermal naevus (green arrows). **(B)** PRAME SL IHC showing +4 PRAME positivity (red nuclear labelling) of atypical melanocytes that are confined to the epidermis with upward pagetoid spread. No PRAME labelling is seen in the underlying dermal naevus (green arrows) which is in favour of an underlying naevus. **(C)** PRAME/Melan A DL IHC illustrates two melanocyte populations: the LM junctional component featuring double-labelled atypical melanocytes (white arrows) and the underlying dermal nevus exhibiting solely Melan A brown cytoplasmic labelling (green arrows).

Several images Figures 5–9 were selected from the study to highlight superimposed DL and SL with various morphological H&E appearances in order to assess the potential utility and application of the novel DL as a potentially useful adjunct within margin assessment investigations.

## Discussion

Several instances of PRAME DL IHC have been described in the literature, combining PRAME with cytoplasmic Melan A or HMB45 [11–15]. Three of these studies ran parallel PRAME SL IHC as the control for the novel DL, correlating results through semi-quantitative scoring [11, 13, 14]. Therefore, the current study design and methodology align with previous research, allowing for comparisons to laboratory aspects of PRAME DL and obtained results to established methods (PRAME SL IHC).

Interpretation and scoring of PRAME IHC results with respect to both distribution and intensity is currently lacking in standardisation. As a result, greater consideration within the study has been made towards the PRAME IHC scoring criteria in which to assess both PRAME SL within SMMS and by extension, assessment of the Novel DL. Many studies, including those which have explored PRAME DL techniques have utilized the >75% threshold for positivity, irrespective of intensity, established by Lezcano et al [7–15]. This scoring method is used in the current study for its high correlation to LM, whilst providing a simpler and direct comparison of results to prior studies [11–15].

While an additional scoring criterion incorporating PRAME labelling intensity has been described in the literature, it was not included in this study due to anticipated technical challenges which could not be controlled for given the retrospective nature of the study [16]. In particular, PRAME intensity scoring has only been described for SL IHC methods, not factoring in the potential for chromogen overlap within a DL protocol, making the interpretation of weaker intensity scoring potentially difficult, further complicating analysis [16, 18]. This is further confounded by the often observed and seemingly inherent variability of intensity for PRAME, within practice - constituting a known pitfall of PRAME IHC [18].

To our knowledge, the literature lacks instances of using a red chromogen for PRAME and a brown chromogen for Melan A in demonstrating DL for PRAME/Melan A [11, 13–15]. Previous studies opted for the reverse chromogen set-up (Figure 1A), citing potential interpretation difficulties with native melanin pigment as a result [13, 14]. In contrast, this study employed and explored the use of red chromogen for PRAME in conjunction with brown chromogen for Melan A, in order avoid challenges due to melanin pigment. However, it is evident that there are accompanying interpretation concerns in relation to the current chromogen arrangement used in the study as shown in Figures 4, 9. These examples, while nuclear PRAME labelling can still be seen, the intense darker background produced by the brown chromogen kit and the cytoplasmic labelling of dense melanocytic nests by Melan A, produce a challenging image for interpretation. In these instances, the reverse chromogen arrangement could be beneficial for pathologists as an alternative (Figure 1A).

Further protocol optimisation could also be explored towards the reduction of the IHC Melan A cytoplasmic component, through the dilution of primary antibodies used, which may also help facilitate sensitivity to PRAME scoring based on intensity, making it easier to discern lower intensity PRAME labelling within the DL method. However, given the use of a closed IHC system and RTU antibodies, this constitutes as a limitation to current study design—setting the basis for future technical exploration through other IHC systems.

The agreement between DL and SL slides was 96.1% (49/51) for PRAME and 100% for Melan A (51/51). The loss of signal focused on PRAME within the DL, with one +1 slide (1/5) and another +2 slide (1/2) being discordant within positive margin specimens only. Reasons for the loss of PRAME in the two discordant positive margin DL slides could be due to a number factors, from the pre-analytical processing of tissues, to the aforementioned technical considerations and the DL protocol itself. Furthermore, the weaker +1 and +2 PRAME labelling in the SL benchmark, indicates the presence of a smaller number of LM cells, which could be harder to detect through the novel DL protocol given that the proportionality of malignant (LM) to benign (background melanocytic hyperplasia) melanocytes will shift further towards the latter. Nevertheless, most examples of weaker (Figure 3) +1 (4/5) PRAME labelling within positive margins was detected successfully in the DL demonstrating high analytical sensitivity.

The total PRAME sensitivity in this study was 78.4% (40/51) utilising a >75% threshold, with a further breakdown showing 91.3% sensitivity in de-bulk specimens and 67.9% at morphologically positive margins. A comparable study by De Wet, Du Plessis, and Schneider (2022) reported a similar PRAME IHC sensitivity profile of 63% within SE LM margin biopsies, using the same criteria of >75% positivity threshold for tumor nuclei [7]. In addition, they emphasized the need for further investigation into the significance of lesser PRAME labelling (+1, +2, +3), raising questions in regards to the suitability of the >75% threshold for PRAME “diffuse” positivity, specifically within SE LM margins. These sentiments are echoed in the current study findings whereby a significant number of PRAME SL slides (7/28) showed intermediate or non-diffuse (classified as negative) PRAME positivity within the margins. The heterogeneous nature of LM, combined with a background of benign atypical melanocytic hyperplasia in sun-damaged skin, may necessitate alternative scoring methods for PRAME positivity within SMMS biopsies. Ultimately however, interpretation of PRAME IHC results, irrespective of scoring methods, should be done so within the context of morphological and clinical correlation—allowing for PRAME IHC to be interpreted as “positive/supportive” of LM without the need for the widely adopted “diffuse +4” scoring criteria set out by Lezcano et al [18].

Not all melanomas show diffuse positivity for PRAME and this pitfall extends to rare LM populations in which further PRAME IHC investigations would be invalid and therefore exclusion is advised [7, 8, 18]. Nonetheless, an alternative approach could be to use PRAME/Melan A DL as a “first-line” test in the rare cases whereby the PRAME status is unknown. In this way, adjunct IHC can still be captured through the highly sensitive cytoplasmic component of the Melan A, to help aid in margin assessment. De Wet, Du Plessis, and Schneider (2022) emphasize the importance of IHC as sole reliance on the gold standard H&E and histological criteria may lead to poorer patient management and morbidity [7]. This underscores the need for adjunct IHC as an integral part of LM/LMM margin assessment, advocating for tests like PRAME SL IHC and by extension PRAME/Melan A DL IHC. This is particularly relevant as IHC stains are not routinely used in the slow Mohs procedure at St. John’s Dermatopathology Department in which this study has been conducted.

The successful outcomes of this pilot study, providing context and performance for both PRAME SL and PRAME DL within SMMS margin assessment, could pave the way for future investigations. In particular, the prospective use of such stains at the time of initial work-up, with direct pathologist involvement, may become the standard for the diagnosis of SMMS cases.

## Summary Table

### What Is Known About This Subject

• Staged excision techniques such as (slow) Mohs Micrographic Surgery, offer greater margin control over traditional methods.
• Adjunct PRAME Immunohistochemistry (IHC) can help in margin assessment of LM/LMM excision biopsies.
• PRAME can be combined with other markers in novel double-labelling (DL) techniques to further maximise utility.

### What This Paper Adds

• Novel PRAME/Melan A DL IHC is a promising alternative to single labelling counterparts, showing high concordance (96.1%).
• Adjunct PRAME/Melan A DL IHC may aid in the assessment of challenging melanocytic lesions and residual LM within margins.
• Further research on interpreting PRAME in LM/LMM lesions with weak to intermediate labelling at margins would be valuable.

This work represents an advance in biomedical science because it demonstrates successfully, a novel PRAME/Melan A IHC DL technique within LM/LMM SMMS biopsies for aiding in margin assessment.

## Data Availability

The raw data supporting the conclusion of this article will be made available by the authors, without undue reservation.
